# Association of multidisciplinary team meetings and survival in gastric cancer patients: nationwide cohort study

**DOI:** 10.1093/bjsopen/zrag126

**Published:** 2026-08-26

**Authors:** Jonathan Engborg, Jakob Hedberg, Mats Lindblad, Gustav Linder

**Affiliations:** Department of Surgical Sciences, Uppsala University, Uppsala, Sweden; Department of Surgery, Höglandssjukhuset, Region Jönköpings Län, Eksjö, Sweden; Department of Surgical Sciences, Uppsala University, Uppsala, Sweden; Department of Upper Abdominal Diseases, Karolinska University Hospital, Stockholm, Sweden; Department of Clinical Science, Intervention and Technology, Division of Surgery, Karolinska Institutet, Stockholm, Sweden; Department of Surgical Sciences, Uppsala University, Uppsala, Sweden

**Keywords:** gastric adenocarcinoma, tumour board, prognosis, population based quality registry

## Abstract

**Background:**

Since 2015, multidisciplinary team (MDT) discussion is recommended for all Swedish patients diagnosed with gastric cancer. However, few studies have explored the association of MDT discussion with survival.

**Methods:**

This study included all Swedish patients diagnosed with gastric adenocarcinoma between 2006 and 2021. Multivariable logistic regression was used to investigate the probability of receiving treatment recommendation at an MDT meeting, whereas the association between receiving an MDT treatment recommendation and overall survival was examined using Cox regression and Kaplan–Meier methods.

**Results:**

Of 7813 patients, 5279 (68%) were discussed at an MDT meeting. Advanced age (80–89 years; odds ratio (OR) 0.30; 95% confidence interval (c.i.) 0.21 to 0.43) and lower performance status (Eastern Cooperative Oncology Group performance status 1; OR 0.68; 95% c.i. 0.56 to 0.81) were associated with a lower probability of receiving an MDT treatment recommendation. Clinical disease stages II and III, compared with stage I, were associated with a higher probability of an MDT discussion (OR 1.38 (95% c.i. 1.10 to 1.75) and 1.42; (95% c.i. 1.09 to 1.85), respectively), as was a later year of diagnosis (OR 1.37; 95% c.i. 1.35 to 1.40). In adjusted survival analysis with multivariable Cox regression, MDT discussion was associated with better survival (hazard ratio 0.89; 95% c.i. 0.83 to 0.95). In addition, median survival was longer in the group with than without MDT treatment recommendations (12.0 *versus* 5.9 months).

**Conclusion:**

Receiving a treatment recommendation at an MDT meeting was associated with better survival in patients with gastric adenocarcinoma. Patients with advanced disease and older age were less likely to receive an MDT treatment recommendation but, if they did, it was associated with better survival.

## Teaser

This nationwide Swedish cohort study look at the association between multidisciplinary team (MDT) assessment and overall survival in patients with gastric adenocarcinoma. Patients discussed at MDT meetings showed improved overall median survival. Older patients and those with poorer performance status were less likely to be presented at an MDT meeting but, despite lower presentation rates, MDT assessment conferred survival benefits even in older patients and those with advanced-stage disease.

## Introduction

Gastric cancer is the fifth most common cancer globally, with a significant worldwide impact and approximately one million new cases detected annually^1^. Most cases of gastric cancer occur in Asia (71.4%), whereas incidence rates in Western countries have been declining in recent decades. This decline is most likely attributed to a lower prevalence of *Helicobacter pylori* infection, reduced tobacco smoking, and decreased consumption of carcinogenic foods. In Sweden, gastric cancer incidence rates have decreased to one-third the levels in the 1980s, with approximately 800 patients diagnosed in 2021. However, gastric cancer remains a grave diagnosis, with approximately 80% of patients succumbing to the disease, often due to late-stage diagnosis and aggressive tumour biology. Surgical resection is essential for a potential cure, and the addition of perioperative and adjuvant oncological treatments has improved survival outcomes^2,3^.

In Sweden, most cancer care is given within the public health system^4^. A decreasing incidence of gastric cancer, combined with increasingly complex multimodal treatment strategies, has prompted both the centralization of surgical treatment and the introduction of multidisciplinary team (MDT) meetings following diagnosis^5^. MDT meetings are considered an important component of clinical decision-making for various malignancies, fostering collaborative and educative discussions among specialists from different level hospitals, disciplines, and professions^6^.

In Sweden, the implementation of MDT meetings occurred in parallel with the progressive centralization of surgical and oncological cancer care, initially as a non-mandatory recommendation. In 2015, the National Board of Health and Welfare (Socialstyrelsen) introduced standardized care pathways for cancer diagnostics. The pathways for oesophageal and gastric cancer mandate a regional MDT conference for each patient before treatment recommendation, with the MDTs comprising an oncologist, surgeon, radiologist, and a specialist cancer nurse^7^. Today, MDT services in Sweden are organized according to the six geographical healthcare regions, each anchored by a university hospital responsible for the surgical management of gastroesophageal cancer^8^. Although no randomized trials have investigated the role of MDTs in gastric cancer survival, some studies have reported benefits attributed to MDTs, such as altered treatment plans with improved prognostication^9,10^, increased resection rates^11^, improved time to treatment^12^, increased access to oncological treatment, and higher participation in clinical trials^13^. Although the survival benefit of MDTs in gastric cancer remains unreported, survival benefit has been reported for oesophageal cancer^8^.

This study hypothesized that access to MDTs varies across Sweden and may influence survival. Therefore, the aim of this study was to investigate factors associated with receiving a treatment recommendation at an MDT meeting and its effect on survival in gastric cancer patients.

## Methods

A register-based retrospective cohort study was conducted, including all patients aged 18 years and above diagnosed with gastric adenocarcinoma, including gastric cardia cancers type III (International Classification of Diseases, Tenth Revision (ICD-10) codes C16.0C and C16.1–C16.9) in Sweden between 1 January 2006, and 31 December 2021. Patients were identified through the National Register for Oesophageal and Gastric Cancer (NREV), described in detail elsewhere^14^. The NREV is a comprehensive register with highly valid data^14^ on clinical work-up and treatment recommendations for newly diagnosed patients^15^. The NREV also includes surgical procedural data for resected patients and comprehensive follow-up information, as well as data on oncological treatment. It contains a mandatory variable stating whether the treatment recommendation was made during an MDT meeting (yes/no). A previous validation study^15^ reported that the accuracy of this variable was 93.9%, with Cohen's κ = 0.89, indicating both excellent validity and very low inter-rater variability. This study is presented in accordance with the STROBE statement for cohort studies.

### Statistical analysis

Patients entered the cohort at the time of diagnosis and were followed until death, emigration, or the end of follow-up (2 September 2022), whichever occurred first. All patients were consistently staged using an algorithm according to the eighth edition of the American Joint Committee on Cancer and Union for International Cancer Control tumour node metastasis (TNM) classification system^16^ based on the recorded T, N, and M categories for each patient. Missing data were handled using complete case analysis, but a sensitivity analysis was also conducted using multiple imputation. Binary and categorical variables are presented as percentages and the number of patients per group. Pearson's χ^2^ test was used to evaluate statistically significant differences in categorical baseline characteristics. Patients with missing data in variables included in multivariable models were excluded but retained in descriptive statistics.

In a directed acyclic graph model^17^, the following well established prognostic factors were identified as potentially associated with being discussed at an MDT meeting: sex, age (< 50 years and then in 10-year intervals up to ≥ 90 years), performance status (Eastern Cooperative Oncology Group (ECOG)/World Health Organization (WHO) score 0–4), clinical stage and year of diagnosis (per year or in tertiles of the study period; *Fig. S1*). Variables for region and tumour location (unspecified, proximal, middle, distal) were excluded because they did not meaningfully alter the main findings and were omitted in the interest of parsimony. To assess temporal trends, the study period was divided into two equal intervals: 2006–2013 and 2014–2021.

Possible associations of receiving a treatment recommendation at an MDT meeting were explored using a multivariable logistic regression model. In addition, an MDT treatment recommendation was used as an exposure in survival analyses conducted using Kaplan–Meier curves with log-rank tests and a multivariable Cox regression model. Survival was calculated from date of diagnosis to death from any cause (overall survival). To assess potential regional confounding, a sensitivity analysis was performed including Swedish healthcare region as a covariate in the multivariable Cox regression model. To investigate bias due to missing data, a sensitivity analysis was conducted using multiple imputation by chained equations with 20 imputations for missing values in clinical disease stage and ECOG/WHO performance status using sex, age, year of diagnosis, MDT discussion status, and treatment as predictors. To address the potential for immortal time bias, a sensitivity analysis was performed excluding all patients who died within the median time from diagnosis (date of referral or biopsy) to the date of MDT discussion.

Subgroup analyses were performed with separate Cox models to assess the impact of MDT meetings on patients for whom multidisciplinary management was hypothesized to be less frequent but potentially of considerable importance. These high-risk groups included patients with advanced age (> 80 years), advanced clinical disease stage (stage IV) or poor performance status (ECOG ≥ 3).

All statistical analyses were performed in Stata^®^ version 14.2 (StataCorp, College Station, TX, USA).

Ethics approval was obtained from the regional Ethics Review Board in Stockholm (2013/596-31/3 and 2016/1486-32).

## Results

### Patient characteristics

**Figure 1:**
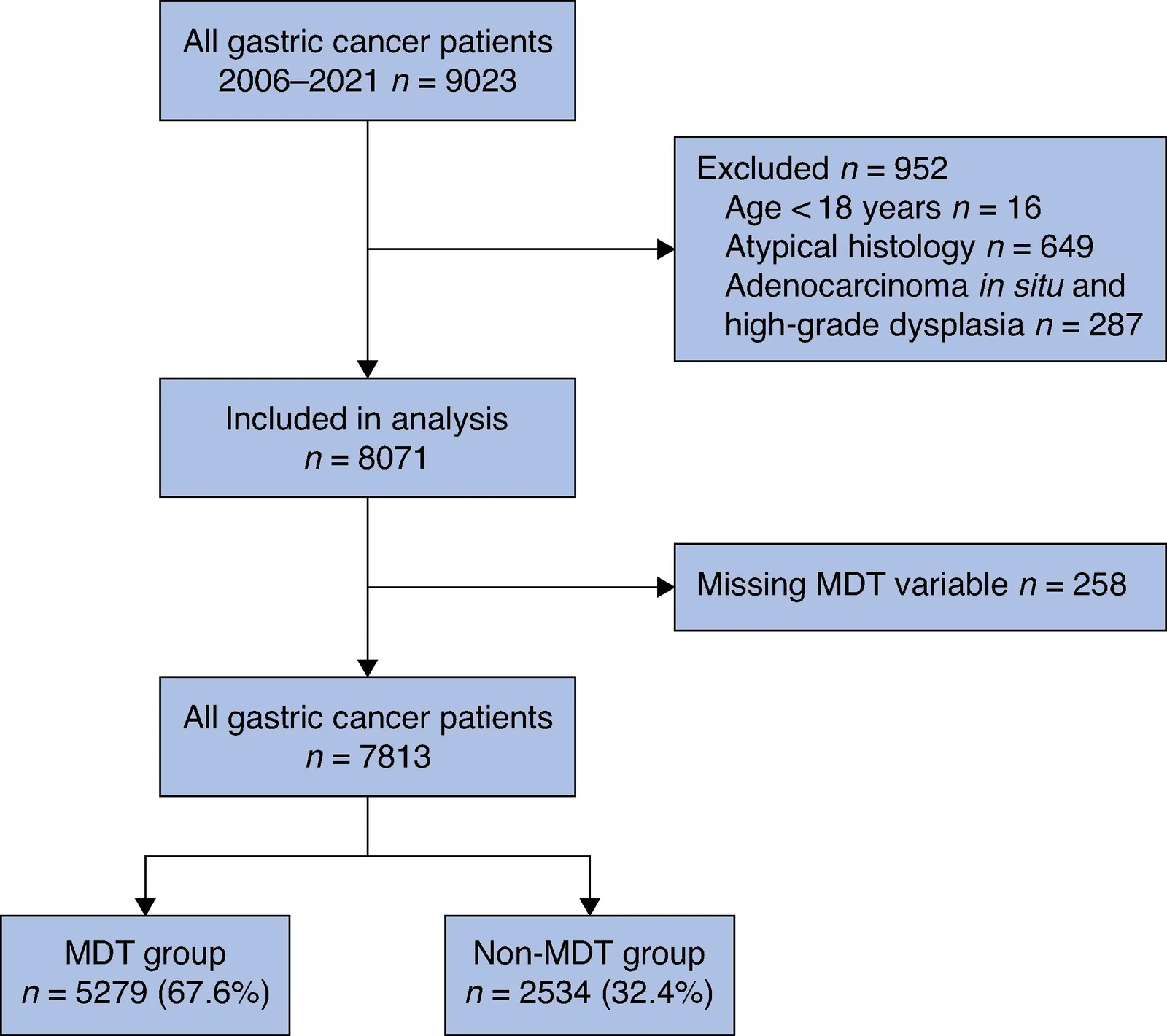
Flow chart of included patients MDT, multidisciplinary team.

**Figure 2:**
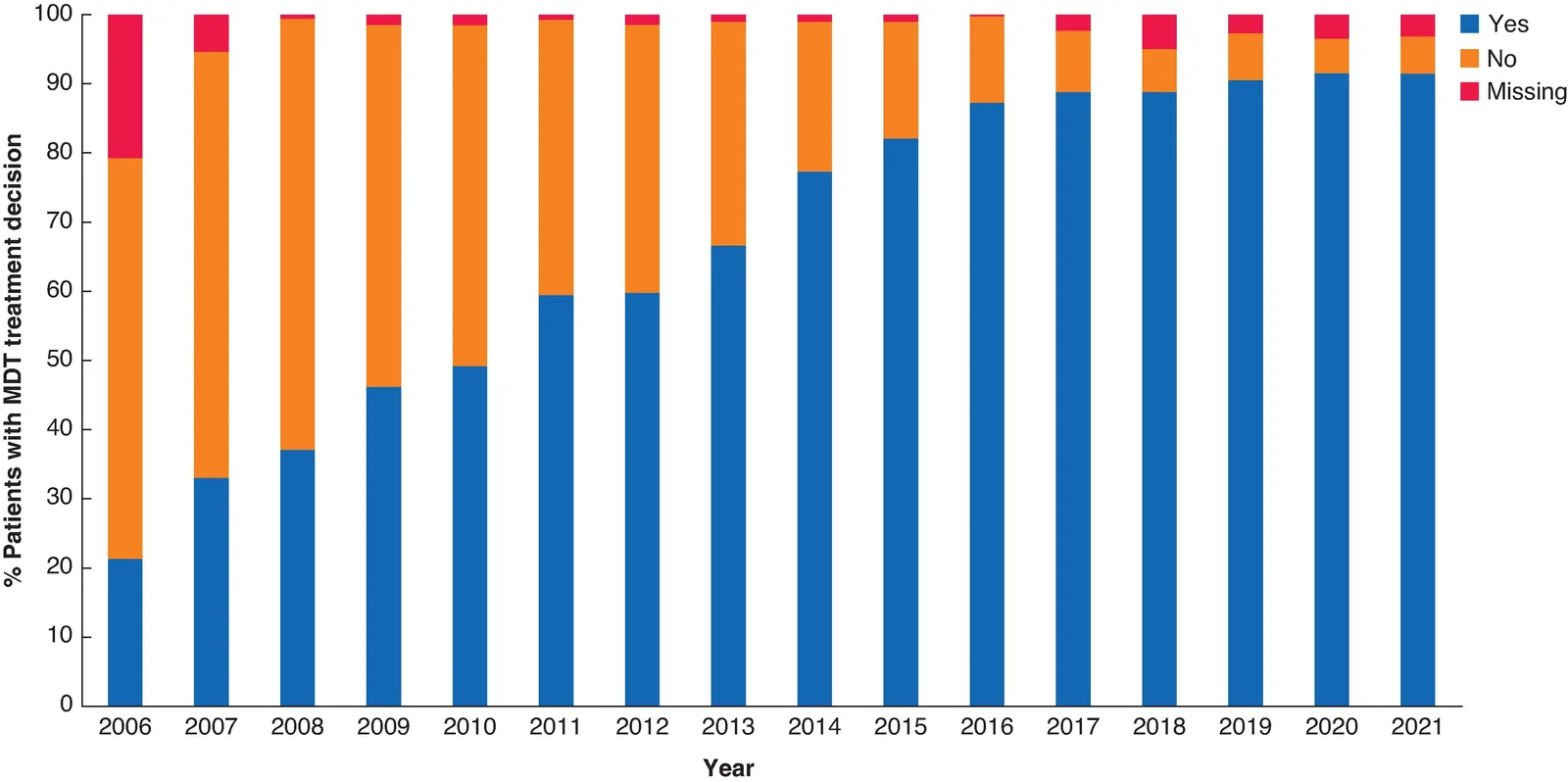
Annual proportions of gastric cancer patients receiving treatment recommendations at an MDT meeting MDT, multidisciplinary team.

Between 1 January 2006 and 30 December 2021, 9023 patients were diagnosed with gastric cancer; 7813 were included in this analysis. Exclusions were due to age <18 years (16 patients), atypical histopathology (for example, gastrointestinal stromal tumours and lymphomas; 649), adenocarcinoma *in situ* and high-grade dysplasia (287), and missing data in the MDT decision variable (258; *Fig. 1*). In the first year of the study, only 21.7% of treatment recommendations were made at an MDT meeting. However, this proportion increased steadily and stabilized at around 90.5% by 2019 (*Fig. 2*). Approximately 9.5% of patients continued not to be discussed at MDT meetings in subsequent years. This group was, in general, older, had advanced disease, worse performance status, and received non-curative treatment recommendations.

In all, 5279 patients (67.6%) were discussed and received treatment recommendations at an MDT meeting. There were differences between patients who were discussed at an MDT meeting and those who were not. A larger proportion of patients in the MDT group were aged < 80 years and had better performance status (ECOG 3–4 9.0% *versus* 17.8% in the MDT and non-MDT groups, respectively) at the time of diagnosis. Most patients in the non-MDT group were diagnosed before 2014 (84.6%), with a marked decrease in this group during the latter half of the study period (to 15.4%). Clinical disease stage differed between the MDT and non-MDT groups primarily in the number of missing values (9.8% *versus* 29.4%, respectively) and the proportion of patients in tumour stages II and III (34.1% *versus* non- 16.6%, respectively). Actual treatment decisions differed as expected because some options, such as neoadjuvant treatment, were not an alternative in the earlier years of the study (*Table 1*).

**Table 1:** Patient characteristics overall and according to receiving treatment recommendation at an MDT meeting

	Total (*n* = 7813)	Treatment decision at MDT meeting		P*
		Yes (*n* = 5279)	No (*n* = 2534)	
**Sex**				0.140
Male	4511 (57.7%)	3078 (58.3%)	1433 (56.5%)	
Female	3320 (42.3%)	2201 (41.7%)	1101 (43.4%)	
**Age category (years)**				< 0.001
< 50	443 (5.7%)	369 (7.0%)	74 (2.9%)	
50–59	755 (9.7%)	592 (11.2%)	163 (6.4%)	
60–69	1650 (21.1%)	1236 (23.4%)	414 (16.3%)	
70–79	2545 (32.6%)	1827 (34.6%)	718 (28.3%)	
80–89	2100 (26.9%)	1128 (21.4%)	972 (38.4%)	
90+	320 (4.1%)	127 (2.4%)	193 (7.6%)	
**Year of diagnosis**				< 0.001
2006–2013	4166 (53.3%)	2022 (38.3%)	2143 (84.6%)	
2014–2021	3648 (46.7%)	3257 (61.7%)	391 (15.4%)	
**ECOG/WHO performance status**				< 0.001
0	2208 (28.3%)	1802 (34.1%)	406 (16.0%)	
1	2625 (33.6%)	1792 (33.9%)	833 (32.9%)	
2	1686 (21.6%)	1006 (19.1%)	680 (26.8%)	
3	746 (9.5%)	405 (7.7%)	341 (13.5%)	
4	180 (2.3%)	70 (1.3%)	110 (4.3%)	
Missing	368 (4.7%)	204 (3.9%)	164 (6.5%)	
**Clinical disease stage**				< 0.001
Stage 0/Tis	55 (0.7%)	26 (0.5%)	29 (1.1%)	
Stage I	1005 (12.9%)	732 (13.9%)	273 (10.8%)	
Stage II	1328 (17.0%)	1073 (20.3%)	255 (10.1%)	
Stage III	892 (11.4%)	726 (13.8%)	166 (6.6%)	
Stage IV	3272 (41.9%)	2207 (41.8%)	1065 (42.0%)	
Missing	1261 (16.1%)	515 (9.8%)	746 (29.4%)	
**Treatment decision**				< 0.001
Upfront surgery	1863 (23.8%)	1223 (23.2%)	640 (25.3%)	
Neoadjuvant chemotherapy and surgery	1424 (18.2%)	1315 (24.9%)	109 (4.3%)	
Palliative care	2564 (32.8%)	1837 (34.8%)	727 (28.7%)	
Best supportive care	1941 (24.8%)	896 (17.0%)	1045 (41.2%)	
Missing	21 (0.3%)	8 (0.2%)	13 (0.5%)	

Values are *n* (%). MDT, multidisciplinary team; ECOG, Eastern Cooperative Oncology Group; WHO, World Health Organization; Tis, tumour *in situ*. *χ2 test or Fisher’s exact test.

### Factors associated with receiving an MDT treatment recommendation

Several factors were significantly associated with the likelihood of receiving a treatment recommendation at an MDT meeting. Patients aged over > 70 years were less likely to be discussed at an MDT meeting than those aged < 50 years. Similarly, patients with a lower performance status (ECOG 1 or higher) were less likely to be discussed. Patients with clinical tumour stages II or III were more likely to be discussed than those with stage I, whereas patients with early (stage 0) or advanced (stage IV) disease had lower likelihoods of being discussed at an MDT meeting. The odds ratio of receiving an MDT recommendation increased by 1.37 per calendar year (*Table 2*).

**Table 2:** Multivariable logistic regression analysis of probability of multidisciplinary team treatment recommendation after diagnosis of gastric cancer (outcome) ( 6288 patients)

	Odds ratio	*P*
**Sex**		
Female	1 (Reference)	
Male	0.98 (0.85, 1.12)	0.765
**Age at diagnosis (years)**		
< 50	1 (Reference)	
50–59	0.93 (0.63, 1.38)	0.320
60–69	0.75 (0.52, 1.06)	0.104
70–79	0.60 (0.42, 0.85)	0.004
80–89	0.30 (0.21, 0.43)	< 0.001
90+	0.13 (0.08, 0.23)	< 0.001
**ECOG/WHO performance status**		
0	1 (Reference)	
1	0.68 (0.56, 0.81)	< 0.001
2	0.51 (0.41, 0.63)	< 0.001
3	0.30 (0.23, 0.40)	< 0.001
4	0.21 (0.13, 0.34)	< 0.001
**Clinical disease stage**		
Stage 0/Tis	0.43 (0.21, 0.84)	0.014
Stage I	1.00 (Reference)	
Stage II	1.38 (1.10, 1.75)	0.006
Stage III	1.42 (1.09, 1.85)	0.010
Stage IV	0.72 (0.59, 0.87)	0.001
Year of diagnosis*	1.37 (1.35, 1.40)	< 0.001

Values in parentheses are 95% confidence intervals. Adjusted odds ratios are shown from a logistic regression model with the outcome being case discussion at a multidisciplinary team meeting. The model was adjusted for sex, age, performance status, clinical disease stage, and year of diagnosis. *Per each additional year from 2006. ECOG, Eastern Cooperative Oncology Group; WHO, World Health Organization; Tis, tumour *in situ*.

### Survival

**Figure 3:**
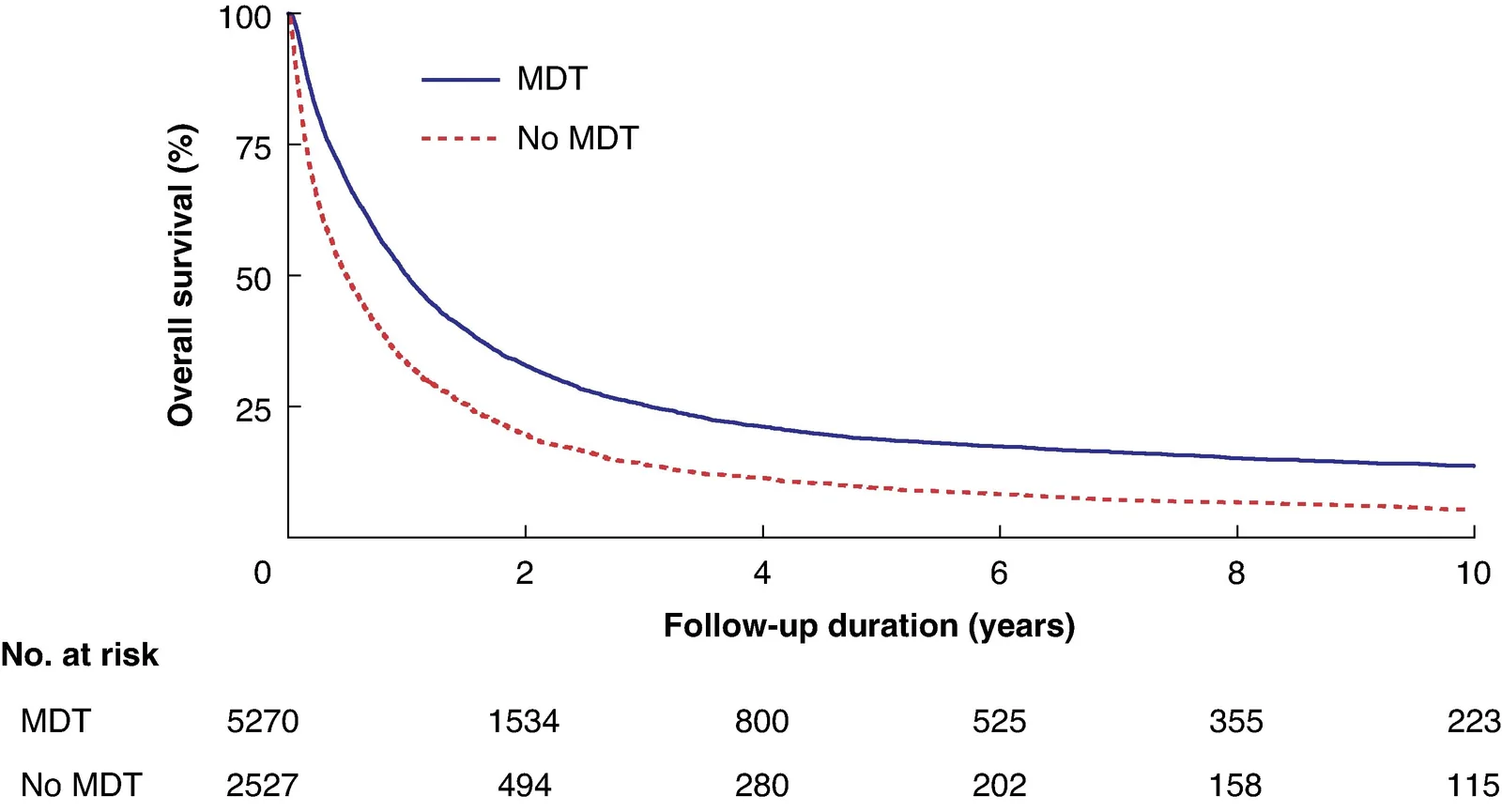
Overall survival of all gastric cancer patients in Sweden according to having received MDT treatment recommendation or not There was a significant difference in overall survival between patients who did and did not receive MDT treatments recommendations (log-rank *P* < 0.001). MDT, multidisciplinary team.

The median overall survival for the entire cohort was 9.6 months. Patients in the MDT group had a median survival of 12.0 months, compared with 5.9 months in the non-MDT group (log-rank *P* < 0.001; *Fig. 3*).

In the multivariable model, hazard ratios (HRs) were adjusted for sex, age, performance status, clinical disease stage, year of diagnosis, and treatment recommendation. Patients whose treatment recommendation was made at an MDT meeting had 11% lower all-cause mortality rate (HR 0.89; 95% c.i. 0.83 to 0.95), whereas men had a 13% higher mortality than women (HR 1.13; 95% c.i. 1.07 to 1.19). Survival also improved slightly over time after 2006 (HR 0.99 per year; 95% c.i. 0.98 to 0.99). Patients who were recommended neoadjuvant chemotherapy followed by surgery had better survival than those who were recommended upfront surgery (*Table 3*).

**Table 3:** Cox proportional hazards model for overall survival in all patients (*n* = 6272)

	Hazard ratio	*P*
**Multidisciplinary team meeting**		
No	1.00 (Reference)	
Yes	0.89 (0.83, 0.95)	0.001
**Sex**		
Female	1.00 (Reference)	
Male	1.13 (1.07, 1.19)	< 0.001
**Age at diagnosis (years)**		
< 50	1.00 (Reference)	
50–59	0.91 (0.79, 1.06)	0.229
60–69	1.11 (0.98, 1.27)	0.114
70–79	1.18 (1.04, 1.35)	0.010
80–89	1.21 (1.06, 1.39)	0.006
90+	1.38 (1.13, 1.69)	0.001
**ECOG/WHO performance status**		
0	1.00 (Reference)	
1	1.34 (1.24, 1.44)	< 0.001
2	1.83 (1.68, 2.00)	< 0.001
3	2.86 (2.56, 3.20)	< 0.001
4	4.03 (3.32, 4.88)	< 0.001
**Clinical disease stage**		
Stage 0/Tis	0.74 (0.53, 1.04)	0.080
Stage I	1.00 (Reference)	
Stage II	1.71 (1.54, 1.89)	< 0.001
Stage III	2.31 (2.06, 2.58)	< 0.001
Stage IV	3.90 (3.50, 4.34)	< 0.001
Year of diagnosis*	0.99 (0.98, 0.99)	< 0.001
**Treatment recommendation**		
Upfront surgery	1.00 (Reference)	
Neoadjuvant chemotherapy and surgery	0.73 (0.66, 0.81)	< 0.001
Palliative treatment	1.61 (1.46, 1.78)	< 0.001
Best supportive care	2.35 (2.12, 2.62)	< 0.001

Values in parentheses are 95% confidence intervals. Analyses were adjusted for sex, age, performance status, disease stage, year of diagnosis, and treatment recommendation. Adjusted hazard ratios are shown. *Per each additional year from 2006. ECOG, Eastern Cooperative Oncology Group; WHO, World Health Organization; Tis, tumour *in situ*.

In a subgroup survival analysis of patients at high risk of not being discussed at an MDT meeting, an MDT treatment recommendation was significantly associated with longer survival. This was true for patients over 80 years of age, for patients with ECOG performance status of ≥ 3, and for patients with stage IV disease (*Tables S1* and *S2*). Covariates associated with poorer outcomes varied across these subgroups. Among patients with all the above risk factors, treatment recommendations made at an MDT meeting remained significantly associated with longer survival (*Table S3*).

To investigate whether receiving a treatment recommendation at an MDT meeting acts as a proxy for higher quality care (that is, rural *versus* academic hospital care), a sensitivity analysis was performed based on the level of MDT (local/regional/national; recorded in the registry starting in 2019). This analysis found no significant differences in survival according to the level of the MDT (*Table S4* and *S5*). When healthcare region was included as a covariate in a sensitivity analysis, the main findings were not meaningfully altered. One region (114 patients, 1.4% of the study population) demonstrated slightly poorer adjusted survival (HR 1.25; 95% c.i. 1.06 to 1.49), although this finding should be interpreted with caution given the small sample size (*Table S6*). The sensitivity analysis addressing the potential for immortal time bias excluded the 292 patients who died within 17 days of the diagnosis, with this time point being the median number of days between diagnosis and MDT discussion, found that the association remained, albeit slightly attenuated (HR 0.92; 95% c.i. 0.87 to 0.99; *Table S7*). In a sensitivity analysis using multiple imputation by chained equations, the association between MDT treatment recommendation and survival remained consistent with the primary analysis (HR 0.88; 95% c.i. 0.83 to 0.94; *Table S8*).

## Discussion

The proportion of Swedish gastric cancer patients receiving treatment recommendations at MDT meetings increased significantly from 21.7% in 2006 to 90.5% in 2019. This study shows that higher age, worse ECOG/WHO performance status, and advanced disease were associated with a lower probability of receiving an MDT treatment recommendation. However, MDT treatment recommendations were associated with better survival not only in the overall cohort, but also specifically in subgroups less likely to be referred for MDT discussion (that is, older patients and patients with low ECOG/WHO performance status and advanced tumour stage). Therefore, age, performance status, and tumour stage alone should not serve as automatic criteria for excluding patients from MDT discussion.

The seemingly low proportion of patients with early-stage disease discussed at MDT meetings could be a result of the European Society of Gastrointestinal Endoscopy recommending MDT presentation for patients after resection in whom endoscopic mucosal resection/endoscopic submucosal dissection show non-radical resection or high-risk characteristics only. In addition, the lower proportion of patients with late-stage disease discussed at MDT meetings could be due to clinicians perceiving that patients would not tolerate active treatment. These findings are further explained in combination with the limited number of slots per weekly regional MDT meeting.

Similar studies on other malignancies have reported better outcomes associated with MDT discussion. For example, a New Zealand study^18^ analysing 509 patients with gynaecological cancers found that MDT meetings resulted in altered treatment plans for 5.9% of patients, but the study did not evaluate whether that translated to improved survival. Similarly, a US study^19^ of non-small-cell lung cancer patients compared outcomes before and after the introduction of MDT meetings and reported improved stage evaluation, better adherence to treatment guidelines, and reduced time from diagnosis to treatment. A larger follow-up study on the same malignancy identified improved quality of life and reduced costs but did not investigate survival^20^. Gaudioso *et al*.^21^ investigated outcomes in 300 lung cancer patients discussed at MDT meetings who were propensity score-matched with 300 patients who were not. In that study, MDT discussion resulted in altered treatment plans in 41% of patients, better adherence to treatment guidelines, and improved overall and cancer-specific survival in stage III and IV disease^21^. The authors attributed the survival benefit in advanced disease to the complex and multimodal treatments these patients often required^21^. Similarly, a Swedish study of patients with synchronous colorectal liver metastases found improved survival in those discussed at a regional MDT meeting (with a liver surgeon present) compared with discussion at a local MDT or no MDT discussion^22^. These studies, and others^9^, underlie the national efforts to increase MDT coverage in Swedish cancer patients, and the improved survival in the present study can be interpreted as reassuring results validating these efforts.

The present study did not evaluate whether MDT meetings improved staging or guideline adherence. However, the higher rate of missing tumour stage data in the non-MDT group suggests that staging accuracy may be better among patients discussed at an MDT meeting. Another potential explanation for better survival is residual case mix factors, such as fewer patients in the MDT group being recommended only best supportive care. Efforts were made to adjust for this in the multivariable survival models for treatment recommendation, and stratified analyses confirmed better survival in the MDT group.

The observed improved survival reported by Gaudioso *et al*.^21^ for lung cancer patients with late-stage disease (III–IV) in the MDT group is consistent with the findings of the present study for high-risk patients (age > 80 years, ECOG performance status 3–4, and stage IV disease). Although adjustments were made for tumour stage, age, and ECOG performance status in the multivariable analysis, the reduced HRs for MDT patients suggest these factors should not necessarily exclude patients from MDT discussions. However, there may be other confounding factors not captured in the present data that may have nudged clinicians towards discussing high-risk patients at MDT meetings. Although the proportion of patients discussed increased, a plateau of 90.5% was reached around 2018. The patient characteristics in this group are described in *Table S9*.

The possibility of confounding effect of care setting on survival warrants consideration because patients discussed at different MDT levels may be disproportionally treated at academic centres, with access to more specialized resources. However, the sensitivity analysis stratified by MDT level did not demonstrate any significant differences, which may reflect an actual effect of the MDT decision-making process itself. Previous studies^23^ have also found geographic and interhospital variations in the proportion of patients discussed at MDT meetings. The present data set could not model random effects by hospital level, but a sensitivity analysis including healthcare region did not alter the primary findings significantly, suggesting that regional variation does not materially confound the results presented here. One small region showed higher mortality, which may reflect random variation given the limited sample size. Furthermore, a previous publication^5^ did not find any difference in treatment recommendation or survival depending on the level of care the diagnosing hospital provided.

The patients not discussed at an MDT meeting comprise a blind spot, because no concrete data are available to explain why the patient was not presented at an MDT meeting. One possibility is that these patients were not motivated to undergo further investigation or treatment, or that complementary investigations (for example, additional radiology or pathology analyses) revealed non-curative disease, leading to a treatment recommendation being made after MDT discussion. This could introduce a bias, because these groups would, on average, have lower survival. Access to experimental treatment or trials may also explain improvements in survival, because some high-risk patients may have gained access to experimental treatment unavailable to patients not discussed at MDT meetings, with the identification of eligibility and possible entry into experimental trials or treatment usually determined at MDT meetings. In addition, with the centralization of curative surgeries, adherence to the latest treatment guidelines is likely higher at academic centres performing the resection surgeries.

This study's strengths include its large patient population and highly validated data. Like other studies, it faces challenges with general healthcare improvements over the study period, such as advances in radiology, an increased emphasis on speedier diagnosis, and early treatment initiation, which coincided with increased adherence to presentation at MDT meetings. These factors may confound the results, because their effects are not necessarily attributable to MDT meetings alone.

Missing data on clinical disease stage also weaken the study results, but may also, to some extent, be viewed as an explanatory mechanism in that patients discussed at an MDT meeting are likely more thoroughly staged and thus have better outcomes. However, a sensitivity analysis using multiple imputation by chained equations yielded results consistent with the primary complete-case analysis, suggesting that missing data in clinical disease stage do not materially bias the study findings. Another weakness of this study is the limited information on co-morbidities; this was addressed by the use of ECOG/WHO status in the registry. More granular data would, of course, be better, but it was not possible to cross-link national registers for this study, which would better capture the full co-morbidity burden that may influence both MDT referral and survival. TNM restaging introduces the Will Rogers phenomenon, where patient migration occurs between groups over time and is impossible to adjust for (for example, before 2010, the N category was defined by the location of positive lymph nodes, but was later based on number of positive lymph nodes). The effect of immortal time bias cannot be fully excluded, but the sensitivity analysis excluding patients who died within the median time to MDT discussion showed attenuated but consistent results with the primary analysis. Finally, improvements in co-morbidity management over time, in relation to gastric cancer treatment, likely correlate with increased MDT participation, because more patients were discussed in the latter part of the study period.

The findings of this study suggest that age, ECOG/WHO performance status, and tumour stage alone should not serve as criteria for excluding patients from MDT discussion. MDT discussion was associated with better survival, although the causal mechanisms underlying these findings cannot be established from the present observational study and warrant further investigation.

## Supplementary Material

zrag126_Supplementary_Data

## Data Availability

The data this study was provided by the Swedish National Register for Oesophageal and Gastric Cancer (NREV) after ethical and registry approval. It can not be shared due to Swedish law, but corresponding extractions from the registry can be provided after ethical and registry approval.
