# Supplementary material for: Association of multidisciplinary team meetings and survival in gastric cancer patients: nationwide cohort study
Source: BJS Open. 2026 Aug 26;10(4):zrag126. doi: 10.1093/bjsopen/zrag126 (PMC13513747; doi:10.1093/bjsopen/zrag126)
Supplement: Supplementary file 1 [file zrag126_supplementary_data.docx]

**Supplementary Materials: Association of multidisciplinary team meetings and survival in gastric cancer patients: nationwide cohort study**

J. Engborg^1,3^, J. Hedberg^1^, M. Lindblad^2^, G. Linder^1^

^1^Department of Surgical Sciences, Uppsala University, Uppsala, Sweden.

^2^Department of Upper Abdominal Diseases, Karolinska University Hospital, Stockholm, Sweden; Department of Clinical Science, Intervention and Technology, Division of Surgery, Karolinska Institutet, Stockholm, Sweden

^3^Department of Surgery, Höglandssjukhuset, Region Jönköpings Län, Sweden

**Corresponding author.**

Jonathan Engborg, MD., PhD-student. Kirurgkliniken, Höglandssjukhuset, Eksjö, Sweden.
ORCID: 0000-0002-6829-8347

E-mail: [Jonathan.engborg@gmail.com](mailto:Jonathan.engborg@gmail.com)
Phone: +46 (0) 76 025 0641

**Supplementary Materials - Index**

| **Supplementary Figures and Tables** |  |
| --- | --- |
| **Table S1** – Cox proportional hazards model for overall survival in all patients with gastric cancer, with subgroup models. | *page 2* |
| **Table S2** – Patient characteristics of patients with age > 80, ECOG 3-4 and IV gastric cancer | *page 3* |
| **Table S3 –** Cox proportional hazards model for overall survival in patients aged >80, ECOG 3-4 and stage IV disease. | *page 4* |
| **Table S4 –** Patient characteristics of patients with recorded level of MDT. | *page 5* |
| **Table S5** – Cox proportional hazards model for overall survival in patients with recorded level of MDT. | *page 6* |
| **Table S6 –** Cox proportional hazards model for overall survival in all patients with tumour location and region included. | *page 7* |
| **Table S7 –** Cox proportional hazards model for overall survival: sensitivity analysis for immortal time bias. | *page 8* |
| **Table S8 –** Cox proportional hazards model for overall survival with multiple imputation by chained equations. | *page 9* |
| **Table S9 –** Patient characteristics of patients diagnosed after 2018. | *page 10* |
| **Figure S1 –** Directed Acyclic Graph from gastric cancer diagnosis to MDT. | *page 11* |

| **Table S1.** Cox proportional hazards model for overall survival in all patients diagnosed with gastric cancer, with subgroup models. | | | | | | | | | | | | | | | |
| --- | --- | --- | --- | --- | --- | --- | --- | --- | --- | --- | --- | --- | --- | --- | --- |
|  | All Patients (same as Table 3) | | |  | Patients with age > 80 | | |  | Patients with ECOG 3-4 | | |  | Patients with Stage IV disease | | |
|  |  | n = 6 272 |  |  |  | n = 1 749 |  |  |  | n = 987 |  |  |  | n = 4 274 |  |
|  | HR | (95% CI) | p |  | HR | (95% CI) | p |  | HR | (95% CI) | p |  | HR | (95% CI) | p |
| **Multidisciplinary team meeting** |  |  |  |  |  |  |  |  |  |  |  |  |  |  |  |
| No | 1.00 | (ref). | - |  | 1.00 | (ref). | - |  | 1.00 | (ref). | - |  | 1.00 | (ref). | - |
| Yes | 0.89 | 0.83-0.95 | 0.001 |  | 0.82 | 0.73-0.93 | 0.001 |  | 0.63 | 0.54-0.74 | <0.001 |  | 0.91 | 0.84-0.98 | 0.012 |
| **Sex** |  |  |  |  |  |  |  |  |  |  |  |  |  |  |  |
| Female | 1.00 | (ref). | - |  | 1.00 | (ref). | - |  | 1.00 | (ref). | - |  | 1.00 | (ref). | - |
| Male | 1.13 | 1.07-1.19 | <0.001 |  | 1.04 | 0.94-1.15 | 0.445 |  | 1.21 | 1.06-1.37 | 0.005 |  | 1.10 | 1.03-1.17 | 0.002 |
| **Age** |  |  |  |  |  |  |  |  |  |  |  |  |  |  |  |
| <50 | 1.00 | (ref). | - |  |  | - |  |  | 1.00 | (ref). | - |  | 1.00 | (ref). | - |
| 50-59 | 0.91 | 0.79-1.06 | 0.229 |  |  | - |  |  | 0.82 | 0.52-1.29 | 0.393 |  | 0.96 | 0.81-1.13 | 0.621 |
| 60-69 | 1.11 | 0.98-1.27 | 0.114 |  |  | - |  |  | 1.08 | 0.74-1.58 | 0.697 |  | 1.07 | 0.92-1.24 | 0.394 |
| 70-79 | 1.18 | 1.04-1.35 | 0.010 |  |  | - |  |  | 1.04 | 0.72-1.50 | 0.839 |  | 1.06 | 0.92-1.23 | 0.427 |
| 80-89 | 1.21 | 1.06-1.39 | 0.006 |  |  | - |  |  | 1.20 | 0.82-1.74 | 0.343 |  | 0.96 | 0.82-1.12 | 0.624 |
| 90+ | 1.38 | 1.13-1.69 | 0.001 |  |  | - |  |  | 1.34 | 0.86-2.09 | 0.201 |  | 0.84 | 0.68-1.03 | 0.095 |
| **WHO/ECOG Status** |  |  |  |  |  |  |  |  |  |  |  |  |  |  |  |
| 0 | 1.00 | (ref). | - |  | 1.00 | (ref). | - |  |  | - |  |  | 1.00 | (ref). | - |
| 1 | 1.34 | 1.24-1.44 | <0.001 |  | 1.21 | 1.01-1.44 | 0.036 |  |  | - |  |  | 1.35 | 1.24-1.48 | <0.001 |
| 2 | 1.83 | 1.68-2.00 | <0.001 |  | 1.73 | 1.44-2.07 | <0.001 |  |  | - |  |  | 1.72 | 1.56-1.90 | <0.001 |
| 3 | 2.86 | 2.56-3.20 | <0.001 |  | 2.83 | 2.31-3.49 | <0.001 |  |  | - |  |  | 2.76 | 2.44-3.12 | <0.001 |
| 4 | 4.03 | 3.32-4.88 | <0.001 |  | 3.91 | 2.90-5.27 | <0.001 |  |  | - |  |  | 3.69 | 3.05-4.45 | <0.001 |
| **Clinical disease stage** |  |  |  |  |  |  |  |  |  |  |  |  |  |  |  |
| Stage 0/Tis | 0.74 | 0.53-1.04 | 0.080 |  | 0.66 | 0.42-1.06 | 0.086 |  | 0.59 | 0.28-1.24 | 0.164 |  |  | - |  |
| Stage I | 1.00 | (ref). | - |  | 1.00 | (ref). | - |  | 1.00 | (ref). | - |  |  | - |  |
| Stage II | 1.71 | 1.54-1.89 | <0.001 |  | 1.55 | 1.31-1.83 | <0.001 |  | 1.72 | 1.27-2.34 | 0.001 |  |  | - |  |
| Stage III | 2.31 | 2.06-2.58 | <0.001 |  | 1.84 | 1.53-2.22 | <0.001 |  | 1.56 | 1.12-2.17 | 0.008 |  |  | - |  |
| Stage IV | 3.90 | 3.50-4.34 | <0.001 |  | 3.38 | 2.87-3.98 | <0.001 |  | 3.66 | 2.75-4.87 | <0.001 |  |  | - |  |
| **Year of diagnosis** | 0.99 | 0.98-0.99 | <0.001 |  | 0.99 | 0.98-1.00 | 0.093 |  | 1.01 | 0.99-1.02 | 0.503 |  | 1.00 | 0.99-1.01 | 0.690 |
| **Treatment recommendation** |  |  |  |  |  |  |  |  |  |  |  |  |  |  |  |
| Upfront surgery | 1.00 | (ref). | - |  | 1.00 | (ref). | - |  | 1.00 | (ref). | - |  | 1.00 | (ref). | - |
| Neoadjuvant chemotherapy & surgery | 0.73 | 0.66-0.81 | <0.001 |  | 1.08 | 0.67-1.75 | 0.741 |  | 0.93 | 0.58-1.48 | 0.753 |  | 1.06 | 0.90-1.25 | 0.486 |
| Palliative treatment | 1.61 | 1.46-1.78 | <0.001 |  | 1.60 | 1.36-1.88 | <0.001 |  | 1.81 | 1.30-2.50 | <0.001 |  | 2.84 | 2.52-3.19 | <0.001 |
| Best supportive care | 2.35 | 2.12-2.62 | <0.001 |  | 1.95 | 1.67-2.29 | <0.001 |  | 2.95 | 2.15-4.04 | <0.001 |  | 4.01 | 3.52-4.55 | <0.001 |

| **Table S2.** Patient characteristics of patients with age > 80, ECOG 3-4 and IV gastric cancer | | | | |
| --- | --- | --- | --- | --- |
|  | Total | Treatment decision at MDT | Treatment decision not made at MDT | p-value |
|  | N=442 | N=155 | N=287 |  |
| Male sex | 217 (49.1%) | 74 (47.7%) | 143 (49.8%) | 0.68 |
| Age Category |  |  |  | 0.083 |
| 80-89 | 360 (81.1%) | 133 (85.8%) | 227 (78.5%) |  |
| 90+ | 84 (18.9%) | 22 (14.2%) | 62 (21.5%) |  |
| Year of diagnosis |  |  |  | <0.001 |
| 2006-2013 | 256 (57.9%) | 51 (32.9%) | 205 (71.4%) |  |
| 2014-2021 | 186 (42.1%) | 104 (67.1%) | 82 (28.6%) |  |
| WHO/ECOG Status |  |  |  | 0.48 |
| 3 | 277 (62.7%) | 108 (69.7%) | 169 (58.9%) |  |
| 4 | 78 (17.6%) | 27 (17.4%) | 51 (17.8%) |  |
| *Missing* | 87 (19.7%) | 20 (12.9%) | 67 (23.3%) |  |
| Clinical disease stage |  |  |  | - |
| Stage IV | 251 (56.8%) | 111 (71.6%) | 140 (48.8%) |  |
| *Missing* | 191 (43.2%) | 44 (28.4%) | 147 (51.2%) |  |
| Treatment Decision |  |  |  | 0.67 |
| Upfront surgery | 10 ( 2.3%) | 3 ( 1.9%) | 7 ( 2.4%) |  |
| Palliative | 70 (15.8%) | 22 (14.2%) | 48 (17.0%) |  |
| Best supportive care | 359 (81.2%) | 130 (83.9%) | 229 (79.8%) |  |
| *Missing* | 3 ( 0.7%) | 0 ( 0.0%) | 3 ( 1.0%) |  |

| **Table S3.** Cox proportional hazards model for overall survival in patients aged >80, ECOG 3-4 and stage IV disease (n = 352). | | | |
| --- | --- | --- | --- |
|  | HR | (95% CI) | p |
| **Multidisciplinary team meeting** |  |  |  |
| No | 1.00 | (ref). | - |
| Yes | 0.73 | 0.57-0.93 | 0.012 |
| **Sex** |  |  |  |
| Female | 1.00 | (ref). | - |
| Male | 1.20 | 0.97-1.49 | 0.093 |
| **Age at diagnosis (years)** |  |  |  |
| 80-89 | 1.00 | (ref). | - |
| 90+ | 0.97 | 0.73-1.29 | 0.834 |
| **WHO/ECOG Status** |  |  |  |
| 3 | 1.00 | (ref). | - |
| 4 | 1.45 | 1.11-1.88 | 0.006 |
| **Year of diagnosis** | 1.01 | 0.99-1.04 | 0.433 |
| **Treatment recommendation** |  |  |  |
| Upfront surgery | 1.00 | (ref). | - |
| Palliative treatment | 1.42 | 0.44-4.55 | 0.558 |
| Best supportive care | 1.59 | 0.51-4.97 | 0.427 |

| **Table S4.** Patient characteristics of patients with recorded level of MDT (introduced 2019). | | | | | |
| --- | --- | --- | --- | --- | --- |
|  | Total | Local MDT | Regional MDT | National MDT | p-value |
|  | N=1 036 | N=128 | N=903 | N=5 |  |
| Male sex | 601 (58.0%) | 82 (64.1%) | 515 (57.0%) | 4 (80.0%) | 0.19 |
| Age Category |  |  |  |  | 0.11 |
| <50 | 84 ( 8.1%) | 6 ( 4.7%) | 77 ( 8.5%) | 1 (20.0%) |  |
| 50-59 | 96 ( 9.3%) | 6 ( 4.7%) | 90 (10.0%) | 0 ( 0.0%) |  |
| 60-69 | 214 (20.7%) | 26 (20.3%) | 188 (20.8%) | 0 ( 0.0%) |  |
| 70-79 | 362 (34.9%) | 38 (29.7%) | 320 (35.4%) | 4 (80.0%) |  |
| 80-89 | 247 (23.8%) | 46 (35.9%) | 201 (22.3%) | 0 ( 0.0%) |  |
| 90+ | 33 ( 3.2%) | 6 ( 4.7%) | 27 ( 3.0%) | 0 ( 0.0%) |  |
| WHO/ECOG Status |  |  |  |  | 0.007 |
| 0 | 363 (35.0%) | 27 (21.1%) | 334 (37.0%) | 2 (40.0%) |  |
| 1 | 327 (31.6%) | 41 (32.0%) | 284 (31.5%) | 2 (40.0%) |  |
| 2 | 174 (16.8%) | 23 (18.0%) | 150 (16.6%) | 1 (20.0%) |  |
| 3 | 114 (11.0%) | 23 (18.0%) | 91 (10.1%) | 0 ( 0.0%) |  |
| 4 | 16 ( 1.5%) | 5 ( 3.9%) | 11 ( 1.2%) | 0 ( 0.0%) |  |
| Missing | 42 ( 4.1%) | 9 ( 7.0%) | 33 ( 3.7%) | 0 ( 0.0%) |  |
| Clinical disease stage |  |  |  |  | 0.001 |
| Stage 0/Tis | 2 ( 0.2%) | 0 ( 0.0%) | 2 ( 0.2%) | 0 ( 0.0%) |  |
| Stage I | 113 (10.9%) | 12 ( 9.4%) | 101 (11.2%) | 0 ( 0.0%) |  |
| Stage II | 227 (21.9%) | 18 (14.1%) | 208 (23.0%) | 1 (20.0%) |  |
| Stage III | 156 (15.1%) | 7 ( 5.5%) | 149 (16.5%) | 0 ( 0.0%) |  |
| Stage IV | 479 (46.2%) | 80 (62.5%) | 395 (43.7%) | 4 (80.0%) |  |
| *Missing* | 59 ( 5.7%) | 11 ( 8.6%) | 48 ( 5.3%) | 0 ( 0.0%) |  |
| Treatment Decision |  |  |  |  | <0.001 |
| Upfront surgery | 202 (19.5%) | 9 ( 7.0%) | 193 (21.4%) | 0 ( 0.0%) |  |
| Neoadjuvant chemo + surgery | 254 (24.5%) | 11 ( 8.6%) | 240 (26.6%) | 3 (60.0%) |  |
| Palliative | 366 (35.3%) | 57 (44.5%) | 307 (34.0%) | 2 (40.0%) |  |
| BSC | 209 (20.2%) | 50 (39.1%) | 159 (17.6%) | 0 ( 0.0%) |  |
| Missing | 5 ( 0.5%) | 1 ( 0.8%) | 4 ( 0.4%) | 0 ( 0.0%) |  |

| **Table S5.** Cox proportional hazards model for overall survival in patients with recorded level of MDT, which the registry introduced in 2019 (n = 958). | | | |
| --- | --- | --- | --- |
|  | HR | (95% CI) | p |
| **Multidisciplinary team meeting level** |  |  |  |
| Local | 1.00 | (ref). | - |
| Regional | 0.90 | 0.72-1.13 | 0.362 |
| National | 2.47 | 0.88-6.97 | 0.088 |
| **Sex** |  |  |  |
| Female | 1.00 | (ref). | - |
| Male | 1.11 | 0.94-1.30 | 0.238 |
| **Age at diagnosis (years)** |  |  |  |
| <50 | 1.00 | (ref). | - |
| 50-59 | 0.64 | 0.41-0.99 | 0.046 |
| 60-69 | 0.92 | 0.64-1.33 | 0.662 |
| 70-79 | 1.07 | 0.75-1.52 | 0.727 |
| 80-89 | 1.07 | 0.73-1.56 | 0.738 |
| 90+ | 1.43 | 0.81-2.54 | 0.215 |
| **WHO/ECOG Status** |  |  |  |
| 0 | 1.00 | (ref). | - |
| 1 | 1.25 | 1.00-1.56 | 0.047 |
| 2 | 1.65 | 1.28-2.13 | <0.001 |
| 3 | 2.57 | 1.90-3.48 | <0.001 |
| 4 | 4.20 | 2.36-7.45 | <0.001 |
| **Year of diagnosis** | 0.97 | 0.87-1.07 | 0.538 |
| **Clinical disease stage** |  |  |  |
| Stage 0/Tis | 3.13 | 0.73-13.3 | 0.123 |
| I | 1.00 | (ref.) | - |
| II | 2.28 | 1.54-3.38 | <0.001 |
| III | 2.31 | 1.51-3.53 | <0.001 |
| IV | 5.88 | 3.96-8.72 | <0.001 |
| **Treatment recommendation** |  |  |  |
| Upfront surgery | 1.00 | (ref). | - |
| Neoadjuvant chemo + surgery | 0.67 | 0.48-0.95 | 0.024 |
| Palliative treatment | 1.43 | 1.01-2.01 | 0.042 |
| Best supportive care | 2.56 | 1.78-3.67 | <0.001 |
|  |  |  |  |

**Table S6.** Cox proportional hazards model for overall survival in all patients with tumour location and region included (n = 6 259).

|  | HR | p-value | 95% CI | |
| --- | --- | --- | --- | --- |
| No MDT | 1 | - | (ref.) | |
| MDT | 0.87 | <0.001 | 0.81-0.93 | |
| Tumour location: |  |  |  |  |
| Unspecified | 1 | - | (ref.) | |
| Proximal | 0.89 | 0.018 | 0.82-0.98 | |
| Middle | 0.89 | 0.002 | 0.82-0.96 | |
| Distal | 0.84 | <0.001 | 0.77-0.91 | |
| Region |  |  |  |  |
| Stockholm | 1 | - | (ref.) | |
| Uppsala | 1.05 | 0.518 | 0.90-1.24 | |
| Södermanland | 0.98 | 0.844 | 0.83-1.17 | |
| Östergötland | 1.09 | 0.232 | 0.94-1.27 | |
| Jönköping | 1.08 | 0.385 | 0.91-1.27 | |
| Kronoberg | 1.16 | 0.081 | 0.98-1.38 | |
| Kalmar | 0.96 | 0.690 | 0.79-1.17 | |
| Gotland | 0.91 | 0.650 | 0.59-1.39 | |
| Blekinge | 0.98 | 0.829 | 0.80-1.20 | |
| Skåne | 1.16 | 0.003 | 1.05-1.28 | |
| Halland | 0.88 | 0.165 | 0.74-1.05 | |
| Västra Götaland | 0.94 | 0.231 | 0.86-1.04 | |
| Värmland | 1.05 | 0.603 | 0.88-1.24 | |
| Örebro | 0.99 | 0.915 | 0.83-1.18 | |
| Västmanland | 1.10 | 0.280 | 0.92-1.32 | |
| Dalarna | 1.17 | 0.072 | 0.99-1.38 | |
| Gävleborg | 0.96 | 0.627 | 0.81-1.13 | |
| Västernorrland | 1.25 | 0.010 | 1.06-1.49 | |
| Jämtland | 1.13 | 0.326 | 0.89-1.44 | |
| Västerbotten | 1.14 | 0.077 | 0.99-1.33 | |
| Norrbotten | 1.08 | 0.276 | 0.94-1.24 | |
|  |  |  |  | |
| Female | 1 | - | (ref.) | |
| Male | 1.14 | 0 | 1.07-1.20 | |
| **Age at diagnosis (years)** |  |  |  |  |
| <50 | 1 | - | (ref.) | |
| 50-59 | 0.93 | 0.308 | 0.80-1.07 | |
| 60-69 | 1.11 | 0.122 | 0.97-1.27 | |
| 70-79 | 1.19 | 0.009 | 1.04-1.35 | |
| 80-89 | 1.22 | 0.006 | 1.06-1.40 | |
| 90+ | 1.39 | 0.002 | 1.13-1.70 | |
| **ECOG/WHO Status** |  |  |  |  |
| 0 | 1 | - | (ref.) | |
| 1 | 1.35 | <0.001 | 1.26-1.46 | |
| 2 | 1.84 | <0.001 | 1.69-2.01 | |
| 3 | 2.96 | <0.001 | 2.64-3.31 | |
| 4 | 4.10 | <0.001 | 3.38-4.98 | |
| **Clinical disease stage** |  |  |  |  |
| Stage 0/Tis | 0.74 | 0.084 | 0.53-1.04 | |
| I | 1 | - | (ref.) | |
| II | 1.74 | <0.001 | 1.57-1.93 | |
| III | 2.36 | <0.001 | 2.11-2.65 | |
| IV | 3.97 | <0.001 | 3.56-4.43 | |
| **Year of diagnosis** | 0.99 | <0.001 | 0.98-0.99 | |
| **Treatment recommendation** | | | | |
| Upfront surgery | 1 | - | (ref.) | |
| Neoadj. + surgery | 0.72 | <0.001 | 0.65-0.80 | |
| Palliative treatment | 1.60 | <0.001 | 1.44-1.76 | |
| Best supportive care | 2.33 | <0.001 | 2.09-2.60 | |

**Table S7.** Cox proportional hazards model for overall survival: sensitivity analysis for immortal time bias (excluding deaths within 17 days of index date, n=292).

|  |  | All Patients |  |
| --- | --- | --- | --- |
|  |  | n = 6 091 |  |
|  | HR | (95% CI) | p |
| **Multidisciplinary team meeting** |  |  |  |
| No | 1.00 | (ref). | - |
| Yes | 0.92 | 0.87-0.99 | 0.043 |
| **Sex** |  |  |  |
| Female | 1.00 | (ref). | - |
| Male | 1.13 | 1.07-1.19 | <0.001 |
| **Age at diagnosis (years)** |  |  |  |
| <50 | 1.00 | (ref). | - |
| 50-59 | 0.92 | 0.79-1.07 | 0.272 |
| 60-69 | 1.12 | 0.98-1.28 | 0.084 |
| 70-79 | 1.20 | 1.06-1.37 | 0.006 |
| 80-89 | 1.25 | 1.09-1.43 | 0.002 |
| 90+ | 1.41 | 1.15-1.73 | 0.001 |
| **WHO/ECOG Status** |  |  |  |
| 0 | 1.00 | (ref). | - |
| 1 | 1.34 | 1.25-1.45 | <0.001 |
| 2 | 1.83 | 1.67-1.99 | <0.001 |
| 3 | 2.71 | 2.41-3.04 | <0.001 |
| 4 | 3.64 | 2.95-4.50 | <0.001 |
| **Clinical disease stage** |  |  |  |
| Stage 0/Tis | 0.74 | 0.52-1.04 | 0.082 |
| Stage I | 1.00 | (ref). | - |
| Stage II | 1.71 | 1.54-1.90 | <0.001 |
| Stage III | 2.30 | 2.05-2.58 | <0.001 |
| Stage IV | 3.82 | 3.42-4.26 | <0.001 |
| **Year of diagnosis** | 0.99 | 0.98-0.99 | <0.001 |
| **Treatment recommendation** |  |  |  |
| Upfront surgery | 1.00 | (ref). | - |
| Neoadjuvant chemotherapy & surgery | 0.73 | 0.66-0.81 | <0.001 |
| Palliative treatment | 1.65 | 1.50-1.83 | <0.001 |
| Best supportive care | 2.27 | 2.04-2.53 | <0.001 |

**Table S8.** Cox proportional hazards model for overall survival with multiple imputation by chained equations (20 imputations) for missing values in clinical disease stage, WHO/ECOG status using sex, age, year of diagnosis and treatment recommendation.

|  |  | All Patients |  |
| --- | --- | --- | --- |
|  |  | n = 7 776 |  |
|  | HR | (95% CI) | p |
| **Multidisciplinary team meeting** |  |  |  |
| No | 1.00 | (ref). | - |
| Yes | 0.88 | 0.83-0.94 | <0.001 |
| **Sex** |  |  |  |
| Female | 1.00 | (ref). | - |
| Male | 1.09 | 1.04-1.15 | <0.001 |
| **Age at diagnosis (years)** |  |  |  |
| <50 | 1.00 | (ref). | - |
| 50-59 | 0.92 | 0.80-1.05 | 0.218 |
| 60-69 | 1.11 | 0.98-1.26 | 0.104 |
| 70-79 | 1.16 | 1.02-1.30 | 0.020 |
| 80-89 | 1.15 | 1.02-1.31 | 0.028 |
| 90+ | 1.16 | 0.98-1.38 | 0.086 |
| **WHO/ECOG Status** |  |  |  |
| 0 | 1.00 | (ref). | - |
| 1 | 1.34 | 1.25-1.44 | <0.001 |
| 2 | 1.73 | 1.59-1.87 | <0.001 |
| 3 | 2.61 | 2.36-2.90 | <0.001 |
| 4 | 3.89 | 3.17-4.77 | <0.001 |
| **Clinical disease stage** |  |  |  |
| Stage 0/Tis | 0.78 | 0.58-1.06 | 0.113 |
| Stage I | 1.00 | (ref). | - |
| Stage II | 1.52 | 1.38-1.67 | <0.001 |
| Stage III | 1.84 | 1.64-2.06 | <0.001 |
| Stage IV | 2.65 | 2.40-2.93 | <0.001 |
| **Year of diagnosis** | 0.99 | 0.99-1.00 | 0.099 |
| **Treatment recommendation** |  |  |  |
| Upfront surgery | 1.00 | (ref). | - |
| Neoadjuvant chemotherapy & surgery | 0.76 | 0.70-0.84 | <0.001 |
| Palliative treatment | 1.95 | 1.78-2.14 | <0.001 |
| Best supportive care | 2.34 | 2.12-2.60 | <0.001 |

| **Table S9.** Patient characteristics of patients diagnosed after 2018. | | | | | |
| --- | --- | --- | --- | --- | --- |
|  | Total | MDT | No MDT | Missing | p-value |
|  | N=1 420 | N=1 295 | N=81 | N=44 |  |
| Male sex | 809 (57.0%) | 745 (57.5%) | 46 (56.8%) | 18 (40.9%) | 0.091 |
| Age Category |  |  |  |  | <0.001 |
| <50 | 116 ( 8.2%) | 108 ( 8.3%) | 4 ( 4.9%) | 4 ( 9.1%) |  |
| 50-59 | 125 ( 8.8%) | 120 ( 9.3%) | 3 ( 3.7%) | 2 ( 4.5%) |  |
| 60-69 | 276 (19.4%) | 267 (20.6%) | 8 ( 9.9%) | 1 ( 2.3%) |  |
| 70-79 | 489 (34.4%) | 448 (34.6%) | 26 (32.1%) | 15 (34.1%) |  |
| 80-89 | 357 (25.1%) | 308 (23.8%) | 28 (34.6%) | 21 (47.7%) |  |
| 90+ | 57 ( 4.0%) | 44 ( 3.4%) | 12 (14.8%) | 1 ( 2.3%) |  |
| WHO/ECOG Status |  |  |  |  | <0.001 |
| 0 | 461 (32.5%) | 452 (34.9%) | 8 ( 9.9%) | 1 ( 2.3%) |  |
| 1 | 416 (29.3%) | 404 (31.2%) | 10 (12.3%) | 2 ( 4.5%) |  |
| 2 | 245 (17.3%) | 222 (17.1%) | 17 (21.0%) | 6 (13.6%) |  |
| 3 | 180 (12.7%) | 145 (11.2%) | 23 (28.4%) | 12 (27.3%) |  |
| 4 | 34 ( 2.4%) | 25 ( 1.9%) | 5 ( 6.2%) | 4 ( 9.1%) |  |
| Missing | 84 ( 5.9%) | 47 ( 3.6%) | 18 (22.2%) | 19 (43.2%) |  |
| Clinical disease stage |  |  |  |  | 0.009 |
| Stage Tis/T0 | 3 ( 0.2%) | 2 ( 0.2%) | 1 ( 1.2%) | 0 ( 0.0%) |  |
| Stage I | 159 (11.2%) | 152 (11.7%) | 6 ( 7.4%) | 1 ( 2.3%) |  |
| Stage II | 299 (21.1%) | 288 (22.2%) | 10 (12.3%) | 1 ( 2.3%) |  |
| Stage III | 199 (14.0%) | 193 (14.9%) | 3 ( 3.7%) | 3 ( 6.8%) |  |
| Stage IV | 642 (45.2%) | 591 (45.6%) | 35 (43.2%) | 16 (36.4%) |  |
| *Missing* | 118 ( 8.3%) | 69 ( 5.3%) | 26 (32.1%) | 23 (52.3%) |  |
| Treatment Decision |  |  |  |  | <0.001 |
| Upfront surgery | 260 (18.3%) | 256 (19.8%) | 4 ( 4.9%) | 0 ( 0.0%) |  |
| Neoadjuvant chemo + surgery | 330 (23.2%) | 324 (25.0%) | 6 ( 7.4%) | 0 ( 0.0%) |  |
| Palliative | 468 (33.0%) | 446 (34.4%) | 20 (24.7%) | 2 ( 4.5%) |  |
| BSC | 320 (22.5%) | 266 (20.5%) | 50 (61.7%) | 4 ( 9.1%) |  |
| Missing | 42 ( 3.0%) | 3 ( 0.2%) | 1 ( 1.2%) | 38 (86.4%) |  |

**Figure S1.** Directed Acyclic Graph from gastric cancer diagnosis to MDT.

Socioeconomic status and comorbidity category was excluded due to lack of data. Tumour location and region omitted due to miniscule effect on other outcomes (see table 5).
